# Role of the AKT signaling pathway in regulating tumor-associated macrophage polarization and in the tumor microenvironment: A review

**DOI:** 10.1097/MD.0000000000041379

**Published:** 2025-01-31

**Authors:** Changming Liang, Song Wang, Chengwei Wu, Jiawei Wang, Lishuai Xu, Senlin Wan, Xu Zhang, Yinfen Hou, Yabin Xia, Li Xu, Xiaoxu Huang, Hao Xie

**Affiliations:** a Department of Gastrointestinal Surgery, The Second Affiliated Hospital of Wannan Medical College, Wuhu, Anhui, China; b Department of Gastrointestinal Surgery, Yijishan Hospital of Wannan Medical College, Wuhu, Anhui, China; c Anhui Province Key Laboratory of Non-coding RNA Basic and Clinical Transformation (Wannan Medical College), Wuhu, Anhui, China.

**Keywords:** AKT signaling pathway, macrophage, polarization, tumor microenvironment

## Abstract

Tumor-associated macrophages (TAMs) are present in and are important components of the tumor microenvironment (TME). TAMs differentiate into 2 functionally distinct morphologies, classically activated (M1)-type TAMs and alternatively activated (M2)-type TAMs, when stimulated by different cytokines. The 2 types of TAMs exhibit distinct properties and functions. M1 TAMs secrete high levels of pro-inflammatory and chemotactic factors, exerting proinflammatory, antitumor effects. Conversely, M2 TAMs alter the extracellular matrix, facilitate cellular immune escape, and stimulate tumor angiogenesis, thereby promoting anti-inflammatory responses and tumor growth. The ratio of M1 TAMs to M2 TAMs in the TME is closely related to the prognosis of the tumor. Tumor cells and other cells in the TME can regulate the polarization of TAMs and thus promote tumor progression through the secretion of various substances; however, polarized TAMs can also act on various cells in the TME through the secretion of exosomes, thus forming a positive feedback loop. Therefore, modulating the phenotype of TAMs in the TME or blocking the polarization of M2 TAMs might be a new approach for cancer treatment. However, the intracellular signaling pathways involved in the polarization of TAMs are poorly understood. The AKT signaling pathway is an important signaling pathway involved in the polarization, growth, proliferation, recruitment, and apoptosis of TAMs, as well as the action of TAMs on other cells within the TME. This paper reviews the AKT signaling pathway in the polarization of TAMs and the regulation of the TME and provides new ideas for tumor immunotherapy.

## 1. Introduction

**Figure 1.**
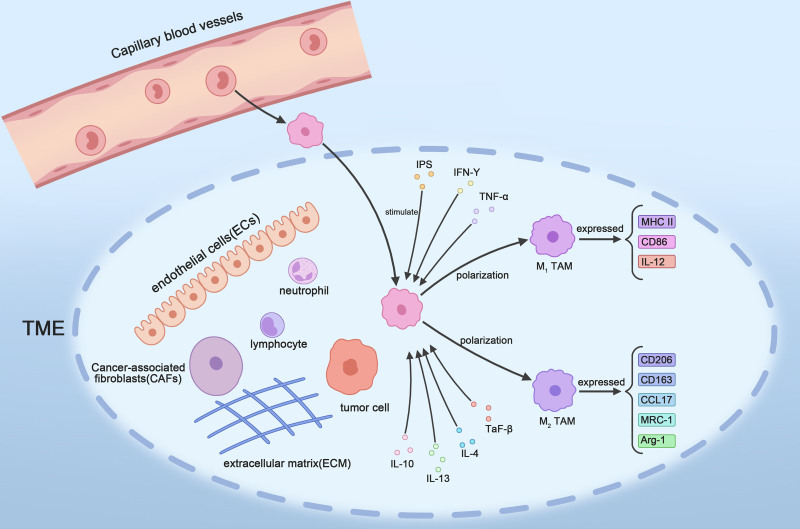
Various components within the tumor microenvironment and how mononuclear macrophages within the blood vessels become tumor-associated macrophages and undergo polarization. As shown in the figure intravascular mononuclear macrophages enter the TME in response to various chemokines and are transformed into TAMs, which polarize into 2 functionally distinct types of TAMs (M1 TAMs and M2 TAMs) in response to different stimulators. TAMs = tumor-associated macrophages, TME = tumor microenvironment.

In many countries and regions worldwide, cancer has become the number 1 cause of death among people older than 70, and its morbidity and mortality rates are increasing rapidly, creating both patient suffering and a heavy burden on society.^[1]^ However, the mechanisms underlying tumor pathogenesis and tumor progression are still poorly understood. In recent years, as research has progressed, tumorigenesis has been found to be a very complex process involving not only the tumor cells themselves but also multiple components within the tumor microenvironment (TME). With the increase in the number of studies concerning crosstalk between tumor cells and various constituents within the TME, tumor-associated immune mechanisms and immunotherapy have become emerging topics of interest.^[2,3]^ The TME refers to the environment in which tumor cells survive in the body and includes, in addition to the tumor cells themselves, immune cells, cancer-associated fibroblasts (CAFs), endothelial cells, a variety of cytokines, the extracellular matrix (ECM), and nutrients. All these components play important roles in tumor growth.^[4]^ Among them, immune cells play important roles in tumor immunosuppression, angiogenesis, growth, invasion and metastasis. Tumor-associated macrophages (TAMs) are the most important type of immune cell in the TME, the most versatile immune cell, and have the greatest impact on tumor progression and prognosis.^[5]^ TAMs are a class of multifunctional and highly plastic cells that differentiate into 2 types with distinct functions and properties – classically activated (M1) TAMs and alternatively activated (M2) TAMs – when stimulated by different factors.^[6]^ M1 TAMs mainly express markers such as MHCII, CD86, and IL-12, which promote inflammatory responses and induce tumor-killing immune responses and play important roles in enhancing immune responses and suppressing tumor development.^[7]^ In contrast, M2 TAMs mainly express markers such as CD206, CD163, CCL17, MRC-1, and Arg-1; serve to inhibit the inflammatory response and play important roles in promoting tumorigenesis, tumor progression, and metastasis^[8]^ (Fig. 1). Many recent studies have shown that the ratio of M1 TAMs to M2 TAMs in the TME is closely related to tumorigenesis, tumor progression, and prognosis. A greater proportion of M2 TAMs in the TME, results in a worse prognosis. Conversely, a greater proportion of M1 TAMs in the TME produces a better prognosis. Therefore, exploring the mechanisms leading to the polarization of TAMs to the M2 type and inducing the transition of M2 TAMs to M1 TAMs in the TME has become an emerging area in tumor research and tumor therapy.^[3]^ Previous studies have shown that the AKT signaling pathway plays an important role in the polarization of TAMs and intercellular crosstalk among the components of the TME as well as in tumorigenesis and development.^[9]^ In this review, we aimed to evaluate the role of the AKT signaling pathway in the polarization of TAMs as well as in intercellular crosstalk in the TME to provide new insights for the immunotherapy of malignant tumors.

## 2. Role of the AKT signaling pathway in the induction of TAM polarization

The Warburg effect is an important feature of tumor metabolism in which tumor cells prefer to increase the rate of glucose uptake and obtain energy through glycolysis rather than through oxidative phosphorylation, even in the presence of sufficient oxygen and fully functioning mitochondria.^[10]^ Although glycolysis does not produce as much adenosine triphosphate (ATP) as oxidative phosphorylation, with only 2 molecules of ATP produced per glucose molecule, glycolysis can produce ATP more rapidly than oxidative phosphorylation.^[11]^ In TAMs, the mode of glucose metabolism is closely related to the phenotype and function of TAMs, with M1 TAMs exhibiting increased glycolysis and decreased oxidative phosphorylation, whereas M2 TAMs exhibit increased oxidative phosphorylation.^[12]^ The final metabolite of glycolysis in animals is lactic acid. The rapid growth of tumor cells leads to a large accumulation of lactate in the TME. In the TME, lactate is both a product of glycolysis and a signaling molecule that can cause M2 polarization of TAMs by activating the AKT signaling pathway. For example, researchers found that lactate secreted by esophageal cancer cells increased the levels of M2 polarization markers on the surface of TAMs, that the levels of phosphorylated AKT and phosphorylated ERK appeared to be markedly elevated in lactate-stimulated TAMs, and that the incorporation of oxalate (a well-recognized inhibitor of lactate) reversed these changes. These experimental results suggest that lactate secreted by esophageal cancer cells stimulates the development of M2 polarization in TAMs through activation of the AKT/ERK pathway.^[13]^ Lactate can stimulate the polarization of TAMs by activating the AKT pathway but also by activating other signaling pathways. For example, in studying a coculture system of MCF-7-TAMR1 cells with TAMs, researchers found that breast cancer cell-derived lactate regulates the polarization of M2 TAMs through the HIF-1α/STAT3 pathway. The stimulatory effect of lactate on the polarization of TAMs was eliminated after treatment with PX-478 (a HIF-1α inhibitor) and S3I-201 (a STAT3 inhibitor) in the coculture system. This finding also suggested that a single substance – lactate – can act through several different pathways to induce the polarization of TAMs.^[14]^

In addition to lactate production by tumor cells through the Warburg effect, the secretion of LGALS9 (encoding the galactoglucan lectin-9 protein, Gal-9) and epidermal growth factor (EGF) by tumor cells is essential for inducing the polarization of TAMs into M2 TAMs. Gal-9 is a secreted protein whose expression is significantly increased in glioblastoma (GBM) with chromosome 10 deletion. Researchers have shown that Gal-9 expression is upregulated in chromosome 10-deficient GBM cells through the activation of AKT and its downstream signaling pathways and that Gal-9 then promotes M2 polarization of TAMs by interacting with the macrophage receptor T-cell immunoglobulin and mucin-domain containing-3 (Tim-3).^[15]^ EGF is a secreted EGF. In colon cancer, EGF secreted by colon cancer cells promotes the polarization of TAMs into M2 TAMs by binding to the EGF receptor (EGFR) on TAMs, which in turn activates the AKT signaling pathway. This study also provides a new strategy for the treatment of colon cancer.^[16]^ Similarly, tumor necrosis factor superfamily member 9 (TNFSF9), which is expressed on various tumor cells, can induce the polarization of TAMs to M2 TAMs by activating the AKT signaling pathway. The migration of TAMs and the metastasis of tumor cells can be promoted by the interaction between tumor cells and immune cells via TNFSF9 and its receptor TNFRSF9.^[17]^ Wu and colleagues reported that in pancreatic cancer, TNFSF9 can promote M2 polarization of TAMs by activating the AKT signaling pathway, which in turn weakens the tumor-killing activity of CD8 + T cells in the TME and contributes to the metastasis of pancreatic cancer.^[18]^

Exosomes are nanovesicles (30–10 nm in diameter) originating from endosomes that transport various cargoes, including RNA, DNA and proteins, into recipient cells and thereby play a role in mediating intercellular communication.^[19]^ Exosomes derived from tumor cells can regulate cancer development and progression by altering the phenotype and function of TAMs.^[20–22]^ Xu and colleagues reported that exosomes secreted by carotenoid-treated prostate cancer cells can induce the M2 polarization of TAMs through activation of the PI3K/AKT signaling pathway, altering the immunosuppressive state of the TME and thus promoting tumorigenesis and tumor progression.^[23]^ Among the many kinds of cargo transported by exosomes, miRNAs contained in tumor-derived exosomes have been an emerging area of research, and many studies have shown that tumor-derived exosomal miRNAs can affect the polarization of TAMs through the AKT signaling pathway, which in turn affects tumorigenesis and tumor development. For example, GBM accounts for a high proportion of common malignant brain tumors and is the most common and deadly malignancy of the central nervous system.^[24]^ Researchers have shown that glioblastoma stem cell-derived exosomal miR-6733-5p can induce M2 polarization of TAMs by targeting insulin-like growth factor 2 mRNA-binding protein 3 (IGF2BP3), which in turn activates the AKT signaling pathway.^[25]^ Li and colleagues also found high levels of miR-3591-3p in glioma-derived exosomes, and this high expression of miR-3591-3p can activate the AKT signaling pathway to promote M2 polarization of TAMs by targeting CBLB.^[26]^ PTEN is a phosphatase that dephosphorylates phosphatidylinositol 3,4,5-trisphosphate (PIP3) to phosphatidylinositol 4,5-bisphosphate (PIP2), directly inhibits the PI3K/AKT signaling pathway, and is a direct site of action for a variety of exosomal miRNAs.^[27]^ Researchers have shown that miR-301a-3p is enriched in esophageal squamous cell carcinoma (ESCC)-derived exosomes and directly inhibits PTEN expression, thereby activating the PI3K/AKT signaling pathway to induce M2 polarization of TAMs, which results in increased expression of vascular endothelial growth factor (VEGF) and matrix metalloproteinase 9 in polarized M2 TAMs and, in turn, facilitates tumor angiogenesis.^[28]^ Chen and colleagues reported that miR-222 was significantly enriched in exosomes derived from adriamycin-resistant breast cancer cells and caused M2 polarization of TAMs by directly targeting PTEN, thereby activating the AKT signaling pathway.^[29]^ In addition to miRNAs, circular RNAs in exosomes also play important roles in the polarization of TAMs. circular RNAs are a class of regulatory noncoding RNA molecules that do not contain a covalently closed continuous loop structure with a 5’ terminal cap and a 3’ terminal poly(A) tail.^[30]^ Wang and colleagues reported that circATP2B4 was highly expressed in epithelial ovarian cancer (EOC)-derived exosomes and that circATP2B4 induced M2 polarization of TAMs through activation of the AKT signaling pathway via miR-532-3p signaling, which in turn facilitated EOC cell migration, invasion and epithelial mesenchymal transition (EMT).^[31]^ In addition, lncRNAs within tumor cell-derived exosomes can also affect the polarization of TAMs by acting on the AKT signaling pathway. For example, researchers have shown that the long noncoding RNA HOTAIR (lncRNA HOTAIR) is highly expressed in laryngeal squamous cell carcinoma cells and its exosomes and that exosomal lncRNA HOTAIR activates the AKT signaling pathway by directly acting on and downregulating the expression of PTEN in TAMs, inducing M2 polarization.^[32]^ Wu and colleagues reported that in oral squamous cell carcinoma, the expression of lncRNA UCA1 is increased in cancer stem cell (CSC)-derived exosomes, which promotes the M2 polarization of TAMs by targeting LAMC2 to regulate the AKT signaling pathway within TAMs.^[33]^

In addition, MS4A7-s, a short isoform of MS4A7, and trigger receptor on myeloid cells 1 (TREM1), which is expressed on TAMs, can also regulate the polarization of M2 TAMs through the AKT signaling pathway. Researchers have shown that MS4A7-s can lead to M2 polarization of glioma-associated macrophages through activation of the AKT signaling pathway and drive malignant progression of GBM.^[34]^ Chen and colleagues found that in hepatocellular carcinoma (HCC), knocking down the expression of TREM1 on the surface of TAMs inactivates transmission of the AKT signaling pathway and thus causes the transformation of TAMs from the M2 type to the M1 type, indicating that the AKT signaling pathway may be involved in the process of M2 polarization of TAMs in HCC.^[35]^ Some proteins expressed within TAMs are also involved in the regulation of M2 TAM polarization through the AKT signaling pathway. TAM-specific deficiency of sentrin/sumo-specific protease 3 leads to increased AKT1 phosphorylation, promoting the M2 polarization of TAMs, which in turn leads to increased proliferation and migration of breast cancer cells.^[36]^ Cheng and colleagues reported that tumor necrosis factor α-induced protein 8-like 1 (TIPE1) expression was high in TAMs and negatively correlated with patient survival. Further research revealed that TIPE1 promotes activation of the AKT pathway in TAMs and M2 polarization of TAMs by directly binding to and regulating the metabolism of PIP2 and PIP3.^[37]^ Methylene THF dehydrogenase (MTHFD2) is an enzyme that plays an important role in the cytoplasmic folate pathway in TAMs, and researchers have found that MTHFD2 promotes activation of the AKT signaling pathway, leading to M2 polarization of TAMs through inhibition of the PIP3 phosphatase activity of PTEN.^[38]^ Insulin-like growth factor binding protein-related protein 1 (IGFBP-rP1) is a potential tumor suppressor gene that plays an important role in a variety of cancers, including colorectal and breast cancer. Gao and colleagues reported that IGFBP-rP1 expression was downregulated in endometrial cancer (EC) cells compared with that in normal tissues and that its overexpression inhibited EC cell proliferation and induced apoptosis, whereas AKT overexpression eliminated this effect. In addition, they found that IGFBP-rP1 can directly interact with AKT and inhibit the AKT signaling pathway. The overexpression of AKT in EC cells eliminated the inhibitory effect of IGFBP-rP1 on the M2 polarization of TAMs.^[39]^ Alpha-fetoprotein (AFP) is a single-chain serum glycoprotein belonging to the albumin family that is synthesized primarily by the fetal liver and yolk sac during embryonic development, and its serum concentration rapidly decreases in the months after birth. As a result, normal adults have very low serum concentrations of AFP. Abnormally elevated AFP is common in patients with chronic or active hepatitis, cirrhosis, HCC, genital tumors, and pregnancy, and approximately 70% of patients with HCC have elevated serum AFP levels.^[40]^ Zhang and colleagues reported that AFP is overexpressed in TAMs and can promote the polarization of TAMs to the M2 phenotype by activating the AKT signaling pathway and that this effect of AFP on TAMs can be successfully inhibited by the PI3K/AKT pathway inhibitor Ly294002. Moreover, researchers have also shown that TAM-derived AFP can prevent TAMs from engulfing polystyrene latex beads by activating the PI3K/AKT pathway, whereas tumor-derived AFP can prevent TAMs from engulfing polystyrene latex beads or HCC cells, which suggests that tumor-derived AFP may serve as a novel biotarget for the immunotherapy of HCC patients.^[41]^ The proteins METTL3 and HNRNPA2B1 are important proteins involved in the m6A modification of miRNAs, and researchers have found that knocking down METTL3 and HNRNPA2B1 to decrease their protein expression in TAMs could affect the m6A modification of miR-146b in TAMs, which could significantly reduce the expression of miR-146b and promote activation of the AKT signaling pathway, which contributes to the M2 polarization of TAMs.^[42]^

**Figure 2.**
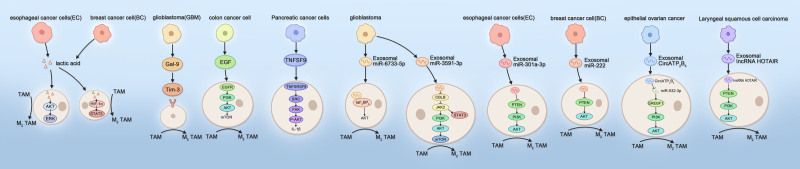
Schematic representation of the mechanism by which multiple different stimuli contribute to M2 polarization of TAMs through the AKT signaling pathway. TAMs = tumor-associated macrophages.

In addition, various components of the TME are involved in regulating M2 TAM polarization through the AKT pathway. SPARC is a cysteine-rich acidic secreted protein that is expressed in a variety of cancer tissues and is involved in a wide range of cell biological behaviors.^[43]^ Deng found that knockdown of SPARC inhibited the M2 polarization of TAMs, and after activation of the PI3K/AKT signaling pathway, the inhibitory effects of SPARC knockdown on the M2 polarization of TAMs and the inhibition of bile duct carcinoma were counteracted.^[44]^ Thymosin β10 (TMSB10) belongs to the β-thymosin family and is an actin-sequestering protein. It has been shown to be overexpressed in most human solid tumors and is involved in the regulation of cancer cell proliferation and metastasis.^[45,46]^ Zeng study revealed that high TMSB10 expression in lung adenocarcinoma promoted the proliferation and M2 polarization of TAMs through activation of the TMSB10-dependent AKT signaling pathway, thereby promoting tumor growth.^[47]^ HSPA12B, an important constituent of the 70 kDa heat shock protein family, is a molecular chaperone that is specifically expressed in tumor-associated endothelial cells and can participate in a variety of cellular protein folding and remodeling processes.^[48]^ Zhou experimentally demonstrated that HSPA12B expression and secretion were significantly elevated in tumor-associated endothelial cells from head and neck squamous cell carcinoma compared with normal human umbilical vein endothelial cells and that aberrantly expressed and secreted HSPA12B was absorbed by TAMs through the expression of oxidized low-density lipoprotein receptor 1, which activates the PI3K/AKT/mTOR signaling pathway in TAMs to induce M2 polarization of TAMs.^[49]^ In addition to these proteins, cytokines, and other proteins within the TME that can influence tumor progression by regulating the polarization of TAMs, the relationship between microbes and tumors has received increasing attention from scientists in recent years.^[50,51]^ Li and colleagues reported that the mucosal microbiota was dysregulated in gastric cancer patients; for example, the abundance of *Propionibacterium acnes* was significantly increased in gastric cancer tissues and promoted the M2 polarization of TAMs through the TLR4/PI3K/AKT signaling pathway.^[52]^ These findings also provide new ideas for the prevention and treatment of gastric cancer (Fig. 2) (Table 1).

**Table 1:** Stimulators that promote M2 polarization of TAM through AKT and other related signaling pathways.

Origin	Stimulators	Signaling pathways	Function	References
Esophageal cancer	Lactate	AKT/ERK	Promote M2 polarization in TAMs	^[13]^
Breast cancer	Lactate	HIF-1α/STAT3	Promote M2 polarization in TAMs	^[14]^
Glioblastoma	Gal-9	Gal-9/ Tim-3	Promote M2 polarization in TAMs	^[15]^
Colon cancer	EGF	EGF/EGFR/AKT	Promote M2 polarization in TAMs	^[16]^
Pancreatic cancer	TNFSF9	AKT	Promote M2 polarization in TAMs	^[17]^
Glioblastoma cell	Exosomal miR-6733-5p	IGF2BP3/AKT	Promote M2 polarization in TAMs	^[25]^
Glioblastoma cell	Exosomal miR-3591-3p	CBLB/AKT	Promote M2 polarization in TAMs	^[26]^
Esophageal squamous cell carcinoma	Exosomal miR-301a-3p	PTEN/PI3K/AKT	Promote M2 polarization in TAMs	^[28]^
Adriamycin-resistant breast cancer	Exosomal miR-222	PTEN/PI3K/AKT	Promote M2 polarization in TAMs	^[29]^
Epithelial ovarian cancer	Exosomal circATP2B4	miR-532-3p/AKT	Promote M2 polarization in TAMs	^[31]^
Laryngeal squamous cell carcinoma	LncRNA HOTAIR	PTEN/AKT	Promote M2 polarization in TAMs	^[32]^
Oral squamous cell carcinoma	LncRNA UCA1	LAMC2/AKT	Promote M2 polarization in TAMs	^[33]^
TAMs	TREM1/ TIPE1/ MTHFD2/ AFP	AKT	Promote M2 polarization in TAMs	^[38]^
Lung adenocarcinoma	TMSB10	AKT	Promote M2 polarization in TAMs	^[47]^
Head and neck squamous cell carcinoma	HSPA12B	PI3K/AKT/mTOR	Promote M2 polarization in TAMs	^[49]^
Gastric cancer tissues	Propionibacterium acnes	TLR4/PI3K/AKT	Promote M2 polarization in TAMs	^[52]^

Abbreviations: AFP = alpha-fetoprotein, EGF = epidermal growth factor, Gal-9 = galactoglucan lectin-9, MTHFD2 = methylene THF dehydrogenase, TAMs = tumor-associated macrophages, TIPE1 = tumor necrosis factor α-induced protein 8-like 1, TMSB10 = thymosin β10, TNFSF9 = tumor necrosis factor superfamily member 9, TREM1 = trigger receptor on myeloid cells 1.

## 3. Role of the AKT signaling pathway in autophagy, apoptosis, infiltration and function of TAMs

Increasing evidence suggests that TAM infiltration and recruitment play crucial roles in tumorigenesis and metastasis.^[53–55]^ Researchers have shown that in breast, pancreatic and bladder cancers, increased infiltration of TAMs is positively correlated with tumor progression and poorer overall patient survival.^[56,57]^ Researchers have shown that the AKT signaling pathway plays an important role in the infiltration and recruitment of TAMs. For example, Li and colleagues reported that high insulin-like growth factor binding protein 7 (IGFBP7) expression was associated with a poor prognosis in gastric cancer patients with the infiltration of TAMs. Further studies revealed that in the TME, increased IGFBP7 secretion by CAFs further promoted the expression and secretion of fibroblast growth factor 2 (FGF2) in CAFs, and FGF2 acted on the fibroblast growth factor receptor (FGFR1) on the surface of TAMs, which in turn activated the PI3K/AKT signaling pathway to promote the infiltration and polarization of TAMs.^[58]^ Wang and colleagues reported that collagen-containing triple helical repeat sequence-1 (CTHRC1) was highly expressed in breast cancer cells and that its high expression was associated with poor prognosis. The results showed that CTHRC1 was associated with the infiltration of TAMs in breast cancer and that CTHRC1 may induce TAM recruitment and M2 polarization by regulating the PI3K/AKT signaling pathway.^[59]^ Other researchers have shown that CTHRC1 expression is upregulated in EC tissues and that CTHRC1 expression is positively correlated with the number of infiltrating TAMs. Mechanistically, CTHRC1 mediates the recruitment of TAMs by activating the integrin β3/PI3K/AKT/CX3CR1 signaling pathway.^[60]^ In addition to promoting the infiltration and recruitment of TAMs through activation of the AKT signaling pathway, researchers have found that many of the components in the TME recruit TAMs by releasing chemokines. For example, upregulation of the expression of the tumor-derived transcription factor forkhead box protein O1 in ESCC can drive the polarization of TAMs and the infiltration of M2 TAMs into the TME, leading to a worse prognosis for ESCC patients. Mechanistically, transcription factor forkhead box protein O1 (+) tumor cells secrete chemokine ligand 20 (CCL20) to promote M2 macrophage recruitment, and this effect can be inhibited by anti-CCL20 antibodies.^[61]^ NADPH oxidase 4 is also abundantly expressed in non-small cell lung cancer (NSCLC), and it can recruit M2 TAMs by activating the ROS/PI3K signaling pathway to produce various cytokines and promote the growth of NSCLC cells.^[62]^

Autophagy is also a key regulatory process in TAMs that both regulates the stability of the intracellular environment and participates in the regulation of multiple specific immune functions.^[63]^ Some researchers have shown that autophagy activation of TAMs in primary HCC promotes the M2 polarization of TAMs.^[64]^ Further studies revealed that the autophagy of TAMs in HCC promotes both the M2 polarization of TAMs and the aggressive biological behavior of HCC cells, and that the AKT signaling pathway plays an important role in this process. For example, Bi and others have shown that miR-210 can promote autophagy in M2 TAMs by targeting the PI3K/AKT/mTOR signaling pathway in HCC. This miR-210-mediated autophagy of M2 TAMs contributes to HCC invasion and proliferation and the inhibition of apoptosis.^[65]^ Additionally, in nasopharyngeal carcinoma (NPC), Yu and colleagues reported that the AKT pathway plays an important role in the autophagy of TAMs. RNF126 is an E3 ubiquitin ligase that has been demonstrated to act as an oncogenic factor in a variety of cancers.^[66]^ They found that after RNF126 entered TAMs through tumor cell exosomes in NPC, it directly interacted with PTEN to decrease its protein expression. The subsequent degradation of PTEN activated the PI3K/AKT pathway and inhibited the autophagy of TAMs, thereby promoting the migration and M2 polarization of TAMs, which in turn promoted the growth and metastasis of NPC.^[67]^

Programmed death ligand-1 (PD-L1) has often been reported to promote immune evasion by tumor cells via depletion of T cells; however, previous studies have focused mainly on the expression of PD-L1 in tumor cells and its inhibitory effect on T cells, and the heterogeneity of PD-L1 expression in other immune cells, particularly TAMs, has not been investigated.^[68,69]^ Xu and colleagues reported that in breast cancer, miR-106b-5p and miR-18a-5p in breast cancer cell-derived exosomes can enter TAMs and promote the expression of PD-L1 in M2 macrophages by activating the PTEN/AKT and PIAS3/STAT3 pathways, thereby promoting the invasion and metastasis of breast cancer cells.^[70]^ Similarly, Yin and colleagues found that in colon cancer (CRC), miR-21-5p and miR-200a in colon cancer cell-derived exosomes are key signaling molecules that mediate the regulatory roles that colon cancer cells play in response to TAMs. Further studies revealed that miR-21-5p and miR-200a exert effects by regulating the PTEN/AKT and SCOS1/STAT1 pathways within TAMs, thereby synergistically inducing M2 polarization and PD-L1 expression in TAMs and leading to a decrease in the activity of CD8 + T cells, which facilitates the immune escape of tumor cells and promotes tumor growth^[69]^ (Table 2). In addition to playing an important role in the expression of PD-L1 within TAMs, the AKT signaling pathway plays an equally important role in the expression of other functions in TAMs. For example, researchers have shown that in HCC, AFP both stimulates the polarization of TAMs toward the M2 phenotype by activating the PI3K/AKT signaling pathway and inhibits the phagocytosis of TAMs. The inhibition of AFP expression could promote the phagocytosis of HCC cells by TAMs.^[41]^ Additionally, in HCC, researchers have shown that VEGF inhibitors exert an inhibitory effect on TAM polarization and function during the interaction between TAMs and tumor cells and that this effect is achieved by inhibiting the VEGFR2/AKT/mTOR signaling pathway.^[71]^

**Table 2:** Stimulators associated with autophagy, apoptosis, infiltration and function of TAMs via the AKT pathway.

Stimulators	Signaling pathways	Function	References
IGFBP7	FGF2/FGFR1/PI3K/AKT	Promote the infiltration and polarization of TAMs	^[58]^
CTHRC1	Integrin β3/PI3K/AKT/CX3CR1	Mediate the recruitment of TAMs	^[60]^
NADPH oxidase 4	ROS/PI3K	Produce various cytokines to recruit M2 TAMs	^[62]^
MiR-210	PI3K/AKT/mTOR	Promote autophagy in M2 TAMs	^[65]^
RNF126	PTEN/PI3K/AKT	Inhibited the autophagy of TAMs	^[67]^
Exosomal miR-106b-5p and Exosomal miR-18a-5p	PTEN/AKT and PIAS3/STAT3	Promote the expression of PD-L1 in M2 macrophages	^[70]^
Exosomal miR-21-5p and exosomal miR-200a	PTEN/AKT and SCOS1/STAT1	Inducing M2 polarization and PD-L1 expression in TAMs	^[69]^

Abbreviations: CTHRC1 = collagen-containing triple helical repeat sequence-1, IGFBP7 = insulin-like growth factor binding protein 7, TAMs = tumor-associated macrophages.

## 4. Crosstalk between TAMs and tumor cells in the TME

Recent studies have revealed that in addition to self-induced alterations in tumor cells, complex crosstalk between tumor cells and components of the TME also plays a large role in the progression of malignant tumors.^[72,73]^ TAMs are important components of the TME, and blood monocyte-derived macrophages are recruited to the TME and polarized into classically activated (M1) and alternatively activated (M2) types, 2 phenotypes with distinct functional roles.^[74]^ M2 TAMs can promote tumor progression by creating an immunosuppressive microenvironment through the production of cytokines, chemokines, and growth factors.^[75]^ In addition, researchers have shown that M2 TAMs can act directly on tumor cells by producing substances such as chemokines or growth factors that promote tumor cell development through crosstalk between TAMs and tumor cells. For example, Chen and colleagues reported that TAMs play an important role in promoting invasive behavior in triple-negative breast cancer (TNBC). High infiltration of M2 TAMs in TNBC tissues was associated with a poor patient prognosis. Mechanistically, researchers have demonstrated that TAMs activate the AKT signaling pathway in tumor cells by secreting chemokine ligand 2 (CCL2), which promotes the expression and nuclear localization of β-catenin and thus promotes EMT and CSC properties of TNBC tumor cells. This effect could be reversed by the knockdown of β-catenin.^[76]^ Messex and colleagues reported that TAMs can affect prostate intraepithelial neoplasia (PIN) progression, that TAMs near the PIN express M2 TAM markers, and that M2 TAMs can promote PIN cell proliferation through the secretion of Spp1, which stimulates the Spp1 receptor expressed on the surface of PIN cells through paracrine secretion, thereby activating the AKT signaling pathway and promoting proliferation.^[77]^ Xie and colleagues reported that the presence of M2 TAMs was associated with a poor prognosis in clear cell RCC (ccRCC) patients. In in vitro experiments, M2 TAMs promoted the proliferation, migration, invasion and EMT of ccRCC cell lines. Through further experiments, researchers have shown that M2 TAMs can secrete chemokine ligand 13 (CXCL13), which in turn activates the AKT signaling pathway in ccRCC cells to promote tumor cell proliferation, migration, invasion, and EMT and that its effects can be inhibited by the use of CXCL13-neutralizing antibodies or AKT inhibitors.^[78]^ Liu and colleagues reported that TAMs significantly accumulate in gastric cancer, that TAMs can secrete matrix metalloproteinases to induce the EMT process in tumor cells by activating the PI3K/AKT/Snail signaling pathway in tumor cells, and that blocking MMP-9 could significantly inhibit the EMT process in tumors and suppress distant metastasis.^[79]^ Exosomes are small double-layered membrane vesicles with a diameter of 30 to 150 nm that can play a crucial role in intercellular crosstalk in the TME by delivering mRNAs, miRNAs, lncRNAs, and other substances.^[19]^ In recent years, M2 TAMs have been shown to engage in intercellular crosstalk with tumor cells by secreting exosomal miRNAs, which can promote the development of tumor cells. For example, Zhang and colleagues found that M2 TAMs in the TME are important drivers of tumor metastasis, and in renal cell carcinoma (RCC), M2 TAMs can secrete exosomes carrying miR-21-5p to act on RCC tumor cells by targeting the noncoding region at the 3’ end of PTEN, which in turn regulates PTEN/AKT signaling to promote the metastasis of RCC cells.^[80]^ Other researchers have shown that miR-183-5p expression is significantly increased in M2 TAMs as well as in their derived exosomes in CRC and that M2 TAMs and their derived exosomes can significantly promote proliferation and invasion and inhibit apoptosis in CRC cells. Further studies revealed that miR-183-5p promotes CRC cell proliferation, invasion, and metastasis by targeting thioesterase superfamily member 4 to activate the PI3K/AKT and NF-kB pathways.^[81]^ In addition, researchers have reported that miRNAs derived from M2 TAM-derived exosomes can both inhibit and promote tumor development. For example, Yao and colleagues reported that in the glioma TME, miR-15a and miR-92a derived from M2 TAM-derived exosomes inhibited glioma cell migration and invasion by activating the PI3K/AKT/mTOR signaling pathway.^[82]^

**Figure 3.**
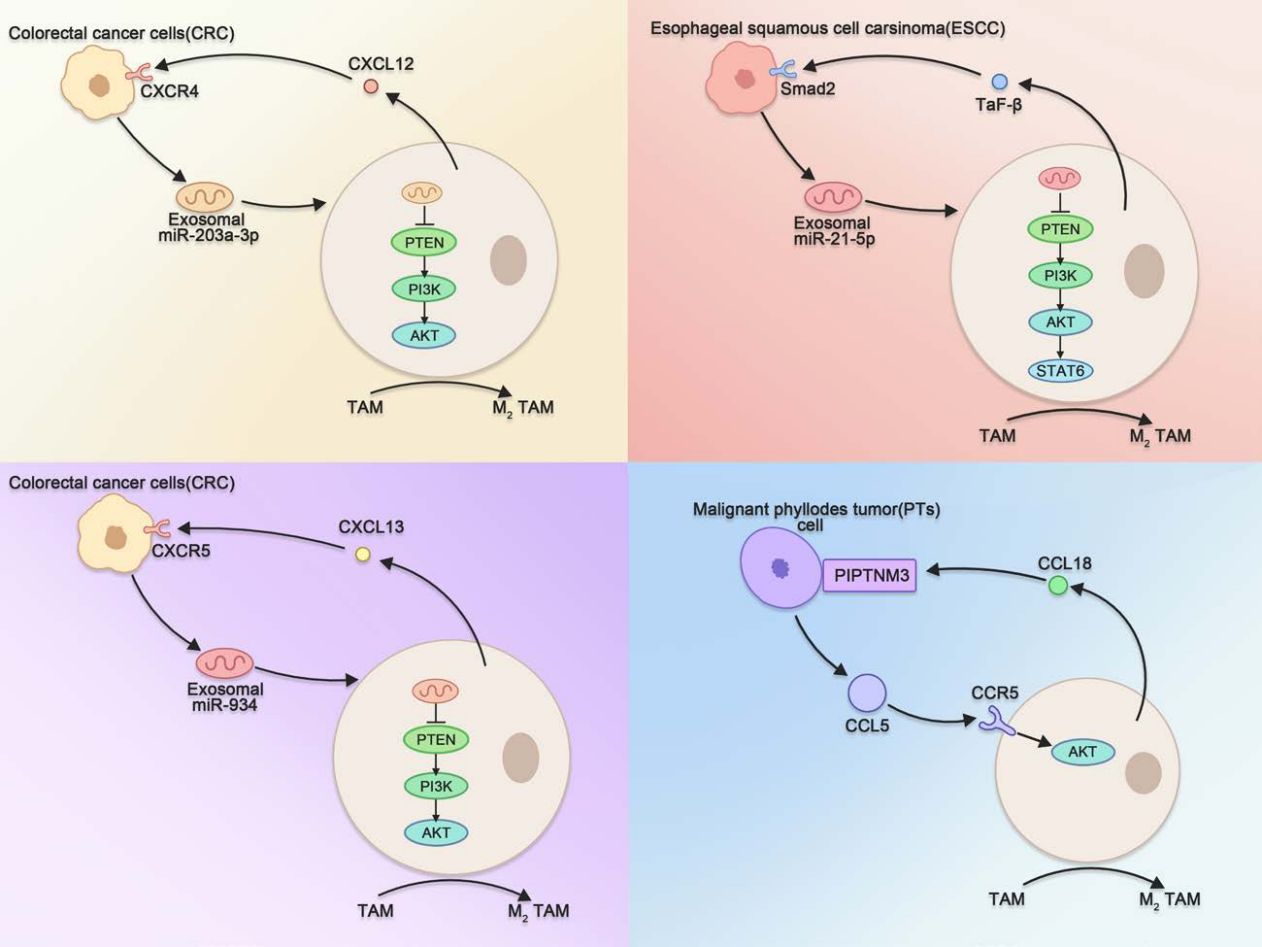
Schematic representation of the positive feedback formed between several tumor cells and TAMs. TAMs = tumor-associated macrophages.

In the TME, tumor cells can act on TAMs by secreting various substances that induce M2 polarization. M2 TAMs can also act on tumor cells by secreting various substances, thus contributing to tumor progression. In recent years, researchers have shown that mutual crosstalk between tumor cells and TAMs creates a positive feedback loop that contributes to malignant tumor progression and metastasis. For example, Pei and colleagues reported that HCT116 colorectal cancer cells can secrete exosomes enriched in miR-203a-3p to TAMs. miR-203a-3p induces M2 polarization of TAMs by targeting PTEN, activating the PI3K/AKT signaling pathway in TAMs and promoting the secretion of CXC chemokine 12 by M2-polarized TAMs. CXC chemokine 12 has a strong chemotactic effect on colon cancer cells and acts on CXC chemokine receptor 4 on the surface of tumor cells to activate the NF-κB p65 pathway, which promotes the further release of exosomes rich in miR-203a-3p from colon cancer cells, thus forming a positive feedback loop.^[83]^ Song and colleagues reported that the content of miR-21-5p in exosomes of ESCC origin was associated with the progression of ESCC. Further studies revealed that after miR-21-5p-rich exosomes were internalized by TAMs, miR-21-5p induced M2 polarization of TAMs through the PTEN/PI3K/AKT/STAT6 signaling pathway; moreover, M2 TAMs might promote EMT in ESCC through the TGF-β/Smad2 signaling pathway. These findings suggest that a positive feedback loop exists between the polarization of M2 TAMs and EMT in the TME in the ESCC; this loop is mediated by the shuttling of miR-21-5p in tumor cell-derived exosomes.^[84]^ Zhao and colleagues reported that miR-934 is aberrantly overexpressed in CRC, particularly in colorectal cancer liver metastases, and is associated with a poor prognosis in CRC patients. Mechanistically, miR-934 in CRC cell-derived exosomes activates the PI3K/AKT signaling pathway by downregulating PTEN expression within TAMs, thereby inducing the M2 polarization of TAMs. Polarized M2 TAMs can activate the CXCL13/CXCR5/NF-KB/p65/miR-934 positive feedback loop in CRC cells by secreting CXCL13.^[9]^ Nie and colleagues reported that the secretion of chemokine ligand 5 by malignant phyllodes tumors (PTs) was associated with a poor prognosis. Chemokine ligand 5 binds to chemokine receptor 5 on the surface of TAMs and activates the AKT signaling pathway within TAMs, thereby recruiting and polarizing TAMs. Subsequently, polarized TAMs release chemokine ligand 18, which further promotes the invasive phenotype of malignant PTs by promoting and maintaining myofibroblast differentiation and invasion. This positive feedback can be blocked by the chemokine receptor 5 inhibitor maraviroc, which prevents the recruitment of peripheral mononuclear macrophages to tumors and significantly inhibits tumor growth^[85]^ (Fig. 3).

In addition, recent studies have shown that crosstalk between tumor cells and other components within the TME can promote the development of tumor drug resistance.^[86,87]^ An increasing number of studies have shown that TAMs can mediate chemotherapeutic resistance in a variety of malignant tumors through crosstalk with tumor cells, and it has also been proposed that blocking the crosstalk between TAMs and tumor cells might be a promising solution to address tumor drug resistance and a new approach to cancer therapy.^[88]^ By detecting macrophage markers in clinical samples from patients, Su and colleagues found that macrophage infiltration might be associated with chemoresistance in gastric cancer. M2 TAMs in the TME were found to inhibit apoptosis and promote resistance to 5-fluorouracil (5-FU) by secreting CXC chemokine ligand 5 (CXCL5) and activating the PI3K/AKT/mTOR signaling pathway in gastric cancer cells. CXCL5 derived from M2 TAMs also act as a chemokine to induce peripheral mononuclear macrophages to enter the TME and promote the formation of a drug-resistant microenvironment in gastric cancer.^[89]^ In CRC, researchers have shown that resistance to 5-FU is also achieved through crosstalk between M2 TAMs and tumor cells. M2 TAMs mediate the resistance of CRC cells to 5-FU through the PI3K/AKT signaling pathway by secreting chemokine ligand 22 (CCL22), and this effect can be inhibited by a CCL22-neutralizing antibody and the AKT inhibitor LY294002.^[90]^ Endocrine therapy is the cornerstone treatment for patients with estrogen receptor-positive breast cancer. However, the effectiveness of endocrine therapy is limited by primary or acquired drug resistance. Qin and colleagues reported that TAMs may play an important role in endocrine resistance in breast cancer, and further experiments revealed that triamcinolone acetonide-resistant MCF-7 cells mediate macrophage polarization to TAMs via the JNK/c-Myc/Arginase-1 pathway. In turn, polarized TAMs promote triamcinolone acetonide resistance by increasing cyclooxygenase-2 (COX-2)/prostaglandin E2 (PGE2) expression and activating the PI3K/AKT/mTOR pathway in MCF-7 cells to promote triamcinolone resistance.^[91]^ Additionally, in estrogen receptor-positive breast cancer, researchers have shown that sodium/glucose cotransporter protein 1 (SGLT1) is highly expressed in endocrine-resistant breast cancer cells, that high expression of SGLT1 promotes glycolysis in endocrine-resistant breast cancer cells, and that lactate produced by from glycolysis in breast cancer cells promotes the M2 polarization of TAMs through the HIF-1α/STAT3 pathway; M2 TAMs promote endocrine resistance in breast cancer via feedback-mediated upregulation of SGLT1 through activation of the EGFR/PI3K/AKT/SGLT1 signaling pathway in breast cancer cells.^[14]^ Li and colleagues reported that endocrine-resistant breast cancer cells are more likely to promote the M2 polarization of TAMs and that M2-polarized TAMs secrete more CCL2 than do M1-polarized TAMs, which activates the PI3K/AKT/mTOR pathway in tumor cells to promote endocrine resistance. CCL2 also contributes to the formation of an endocrine-resistant tumor microenvironment.^[88]^ Researchers found that the gefitinib-resistant HCC827/GR cell line had a greater ability to proliferate and migrate than did the corresponding nonresistant cell line and attracted M2 TAMs by releasing more CCL2, which reduced the antitumor effects of gefitinib treatment by activating the AKT/mTOR pathway in tumor cells.^[92]^ In addition to the M2 TAMs mentioned above, which secrete chemokines to act on tumor cells and mediate their drug resistance through the AKT signaling pathway, TAM-derived exosomes are involved in chemoresistance in many cancers, including EOC.^[93,94]^ Zhu and colleagues reported that TAM-derived exosomes are involved in triggering chemoresistance in EOC cells. Hypoxic EOC cells can promote the recruitment of TAMs and induce their M2 polarization, and M2-polarized TAMs act on EOC cells by secreting miR-223-rich exosomes. miR-223 is internalized and then activates the PI3K/AKT signaling pathway to confer resistance to chemotherapy in EOC cells by targeting PTEN^[95]^ (Table 3).

**Table 3:** Stimulators of TAMs origin that affect tumor cell function through the AKT signaling pathway.

Stimulators	Signaling pathways	Function	References
CCL2	AKT/β-catenin	Promote the EMT and CSC properties of triple-negative breast cancer cells	^[76]^
Spp1	Spp1R/AKT	Promote prostate intraepithelial neoplasia cell proliferation	^[77]^
CXCL13	AKT	Promote ccRCC cells proliferation	^[78]^
Matrix metalloproteinases	PI3K/AKT/Snail	Induce the EMT process in gastric cancer cells	^[79]^
Exosomal miR-21-5p	PTEN/AKT	Promote the metastasis of RCC cells	^[80]^
Exosomal miR-183-5p	PI3K/AKT and NF-kB	Promote proliferation and invasion and inhibit apoptosis in CRC cells	^[81]^
Exosomal miR-15a and exosomal miR-92a	PI3K/AKT/mTOR	Inhibit glioma cell migration and invasion	^[82]^
CXCL5	PI3K/AKT/mTOR	Inhibit apoptosis and promote resistance to 5-FU in gastric cancer	^[89]^
CCL22	PI3K/AKT	Mediate the resistance of CRC cells to 5-FU	^[90]^
COX-2/PGE2	PI3K/AKT/mTOR	Promote triamcinolone acetonide resistance	^[91]^
CCL2	PI3K/AKT/mTOR	Promote endocrine resistance	^[88]^
Exosomal miR-223	PTEN/PI3K/AKT	Confer resistance to chemotherapy in EOC cells	^[95]^

Abbreviations: CCL2 = chemokine ligand 2, CCL22 = chemokine ligand 22, CXCL13 = chemokine ligand 13, CXCL5 = CXC chemokine ligand 5, TAMs = tumor-associated macrophages.

## 5. Perspectives on the role of the AKT signaling pathway in targeted tumor therapy

In recent years, immunotherapy for tumors has become an active research area worldwide, and it is widely recognized as an effective tumor treatment. As an important component of the TME, TAMs play important roles in tumorigenesis and development, and they are also important targets for research on tumor immunotherapy.^[96]^ In addition, cancer therapies targeting TAMs have been increasingly researched and have made considerable progress in recent years.^[97]^ There have been a number of clinical trials of immunotherapies targeting TAMs that have made considerable progress in a variety of cancers^[98–105]^ (Table 4). Many studies have suggested that the ratio of M1 TAMs to M2 TAMs in the TME is closely related to the prognosis of tumors and that the ratio of the 2 in the TME can be altered by either inhibiting the M2 polarization of TAMs or inducing the M1 polarization of TAMs to inhibit tumor growth. For example, Golino and colleagues found a higher M1/M2 ratio in the verteporfin-treated experimental group than in the control group in the CCA YAP/AKT mouse model and an increase in the proportion of activated CD8 + T cells (CD8 + CD25 + and CD8 + CD69+), suggesting that verteporfin suppresses tumorigenesis by affecting the M1/M2 ratio and activating the CD8 + T-cell population within the TME.^[106]^ Therefore, studying the signaling pathways that induce polarization of TAMs is particularly important and clinically relevant. Numerous substances have been found to inhibit tumor growth by acting on the signaling pathways of TAMs to allow their reprogramming. Lu and colleagues found that tamoxifen induced M1 polarization of TAMs by inactivating signal transducer and activator of transcription 6 (STAT6) and blocking the macrophage-specific immune checkpoint src-homology domain 2 (SH2)-containing protein tyrosine phosphatase (SHP)-SHP1/SHP2, which inhibited the migration of pituitary adenoma cells.^[107]^ Fucoidan (FPS1M) is a glycan derived from brown algae that contains pyroxyl groups and sulfate groups, and a large body of research suggests that fucoidan has antitumor activity.^[108]^ Deng and colleagues reported that FPS1M can act as a stimulator of Toll-like receptor 4 (TLR4) to increase macrophage glycolysis and induce M1 polarization of TAMs by activating the TLR4-mediated PI3K/AKT/mTOR signaling pathway. By increasing the infiltration of M1 TAMs in tumor tissues, the ratio of M1/M2 TAMs within the TME was altered, which in turn increased the sensitivity of tumor cells to capecitabine.^[109]^ Recently, the AKT signaling pathway has become heavily researched in tumor immunotherapy because drugs targeting this pathway affect the polarization of TAMs. Saikosaponin d (SSd) is the main active ingredient of saikosaponin, a triterpene saponin that possesses various antitumor activities against lung,^[110]^ liver,^[111]^ and ovarian cancers.^[112]^ Xu and colleagues found that in pancreatic ductal adenocarcinoma, SSd could prevent TAMs from undergoing M2 polarization by downregulating the expression of phosphorylated STAT6 and inhibiting the PI3K/AKT/mTOR signaling pathway, and that SSd treatment also partially abrogated the effects of 740-Y-P (a PI3K activator).^[113]^ These findings suggest that SSd may be a promising new therapeutic agent for treating pancreatic ductal adenocarcinoma. Rheum palmatum is a naturally occurring anthraquinone extracted from rhubarb (*Rheum palmatum* L.) roots that has shown anticancer activity against a variety of cancers both in vivo and in vitro.^[114,115]^ Yin and colleagues found that myricetin induces the transition of M2 TAMs to M1 TAMs via the miR-26a/TGF-β1/AKT axis, which inhibits the growth of HCC and exerts antitumor effects.^[116]^ Myricetin, a natural compound isolated from sterile mycelia, inhibits sphingomyelin synthesis. Jang and colleagues found that myricetin inhibited the PI3K/AKT/mTOR signaling pathway within TAMs, inhibited M2 polarization in TAMs, and subsequently reduced the number of M2 TAMs within tumor tissues in a mouse xenograft tumor model, which inhibited tumor growth by remodeling the TME.^[117]^ AZD8055 is a novel second-generation mTOR inhibitor with excellent selectivity for all PI3K isoforms and other members of the PI3K-related kinase family.^[118]^ Hu and colleagues found that AZD8055 can block the induction of M2 TAM polarization in bladder cancer cells by regulating the PI3K/AKT/mTOR signaling pathway, thus exerting antitumor effects, and that AZD8055 might be a promising mTOR inhibitor for the treatment of bladder cancer.^[119]^ Dihydroartemisinin is a sesquiterpene lactone chemosynthetic extracted from *Artemisia annua*.^[120]^ Xiao and colleagues found that Dihydroartemisinin dose-dependently promotes M1 polarization of TAMs through modulation of the AKT/mTOR signaling pathway by increasing the expression of molecules related to the M1 phenotype while decreasing the expression of molecules related to the M2 phenotype; this in turn inhibits the growth of lung cancer.^[121]^

**Table 4:** Clinical trials related to immunotherapy targeting TAMs in different types of cancers.

Type of cancer	Drug	Target	References
Hepatocellular carcinoma	SNDX-6352	CSF-1R	^[98]^
	BMS-813160	CCR2	^[99]^
	Sorafenib	VEGF2	^[100]^
Breast cancer	Pexidartinib (PLX3397)	CSF-1	^[101]^
	Selicrelumab (RO7009789)	CD40	^[102]^
Lung cancer	Tazemetostat	CCL5	^[103]^
Colorectal cancer	Maglumab (Hu5F9-G4)	CD47	^[104]^
	PF-07265807	TAMK	^[105]^

Abbreviations: CCR2 = chemokine (C-C motif) receptor 2, CSF-1 = colony-stimulating factor 1, CSF-1R = colony-stimulating factor 1receptor, TAMs = tumor-associated macrophages, TAMK = tumor-associated macrophage kinase, VEGF2 = vascular endothelial growth factor.

In addition to the PI3K/AKT signaling pathway, the downstream NF-κB signaling pathway is also an important target for tumor therapy. Verbascoside is a phenylpropanoid glycoside found in a variety of herbal extracts.^[122]^ Ren and colleagues identified Verbascoside as a potential therapeutic agent for ovarian cancer that can induce M1 polarization of TAMs through inhibition of the cellular communication network factor 1 (CCN1)-mediated AKT/NF-kB pathway, which can inhibit tumor cell growth.^[123]^ Tretinoin lactone alcohol is 1 of the main active ingredients of tretinoin, a traditional Chinese medicine (TCM) that can inhibit the growth, invasion, and migration of drug-resistant ovarian cancer cells and reverse cisplatin resistance in ovarian cancer cells by inhibiting the phosphorylation of AKT.^[124,125]^ Le and colleagues reported that the combination of Tretinoin lactone alcohol and cisplatin may promote the transition of TAMs from the M2 phenotype to the M1 phenotype by inhibiting the AKT/NF-kB pathway, thus inhibiting the proliferation of tumor cells.^[126]^ Cheng and colleagues found that β-D-(1→6) glucan (AAMP-A70) decreases CRC cell viability by binding to toll-like receptor 2 on TAMs, thereby increasing the phosphorylation levels of AKT/NF-KB and MAPK and facilitating the transformation of M2 TAMs into M1 TAMs^[127]^ (Table 5).

**Table 5:** Anticancer drugs that work by targeting the AKT signaling pathway.

Anticancer drugs	Mechanism	References
Tamoxifen	Inactivating signal transducer and activator of STAT6 and blocking the SHP-SHP1/SHP2, induce the M1 polarization of TAMs	^[107]^
FPS1M	Activate the TLR4-mediated PI3K/AKT/mTOR signaling pathway, increase the infiltration of M1 TAMs in tumor tissues	^[109]^
Saikosaponin d	Prevent TAMs from undergoing M2 polarization by downregulating the expression of phosphorylated STAT6 and inhibiting the PI3K/AKT/mTOR signaling pathway	^[113]^
Myricetin	Induce the transition of M2 TAMs to M1 TAMs via the miR-26a/TGF-β1/AKT axis	^[116]^
Myricetin	Inhibite the PI3K/AKT/mTOR signaling pathway within TAMs, inhibite M2 polarization in TAMs	^[117]^
AZD8055	Block the induction of M2 TAM polarization by regulating the PI3K/AKT/mTOR signaling pathway in bladder cancer cells	^[119]^
Dihydroartemisinin	Promote the M1 polarization of TAMs through the modulation of the AKT/mTOR signaling pathway	^[121]^
Verbascoside	Induce the M1 polarization of TAMs through inhibition of CCN1-mediated AKT/NF-kB pathway	^[123]^
AAMP-A70	Bind to TLR2 on TAMs, thereby increasing the phosphorylation levels of AKT/NF-KB and MAPK and facilitating the transformation of M2 TAMs into M1 TAMs	^[127]^

Abbreviations: CCN1 = cellular communication network factor 1, FPS1M = fucoidan, SHP = Src homology-2 domain-containing protein tyrosine phosphatase, STAT6 = signal transducer and activator of transcription 6, TAMs = tumor -associated macrophages, TLR2 = toll-like receptor 2.

In recent years, many studies have demonstrated that tumor cells can alter the tumor microenvironment by delivering exosomes, thereby causing the tumor microenvironment to shift and promote tumor growth. Therefore, exosomes released by tumor cells and some of the substances contained in exosomes can also serve as important targets for tumor therapy. For example, Peng and colleagues reported that exosomes released from prostate cancer cells induced the polarization of TAMs to M2 TAMs by activating the AKT and STAT3 signaling pathways. The exosome biogenesis inhibitor GW4869 inhibited the release of exosomes from tumor cells, thus inhibiting the exosome-induced polarization of TAMs to the M2 phenotype and suppressing the progression of prostate cancer in cell-based models. GW4869 was found to play an important role in the treatment of prostate cancer metastasis.^[128]^ Researchers have also found that some drugs can inhibit cancer progression by decreasing the levels of certain substances within tumor-derived exosomes. Modified Jianpi Yangzheng decoction, a TCM used to “strengthen the spleen” and “nourish Zhen Tang,” has shown great therapeutic potential in the prevention and treatment of gastric cancer. Wu and colleagues reported that pyruvate kinase M2 (PKM2) within exosomes derived from gastric cancer cells can be internalized by TAMs, resulting in upregulation of early growth response factor 1 (Egr-1) within TAMs and leading to the M2 polarization of TAMs and the promotion of tumor progression. Additionally, Modified Jianpi Yangzheng decoction can directly induce apoptosis in gastric cancer cells by inhibiting the PI3K/AKT/mTOR signaling pathway and can also prevent TAMs from undergoing M2 polarization by decreasing the amount of PKM2 in tumor cell-derived exosomes, thus playing an antitumor role.^[129]^ In recent years, nanoplatform-mediated chemotherapeutic drug delivery has facilitated the development of clinical cancer therapies. Liposomes have been used as popular drug delivery systems because of their self-assembly properties, ability to encapsulate water-soluble and lipophilic drugs, and excellent pharmacokinetic characteristics.^[130,131]^ Yang and colleagues combined BRD4-degrading protein hydrolysis-targeted chimera (PROTAC) ARV-825 with a composite micelle (SPP) consisting of substance P (SP) peptide-modified poly(ethylene glycol)-poly(D,L-lactic acid) (SP-PEG-PDLLA) and methoxy poly(ethylene glycol)-poly(D,L-lactic acid) (mPEG-PDLLA, PP). Thus, a therapeutic nanosystem (SPP-ARV-825) that can penetrate the blood–brain barrier to target brain tumors was constructed. This nanosystem can overcome the inefficiency of chemotherapeutic drugs in crossing the blood–brain barrier (BBB), a major challenge in antiglioma therapy. It has been experimentally confirmed that this system can achieve good antitumor effects in glioma treatment by suppressing the proliferation of glioma cells, inducing apoptosis, and suppressing the M2 polarization of TAMs.^[132]^ Li and colleagues fused CD47-expressing tumor exosomes with tumor-targeting peptide cRGD (cyclic arginine-glycine-aspartic acid)-modified liposomes (miRNA-497/TP-HENPs) to form bioinspired hybrid nanoparticles for the codelivery of miR-497 and tretinoin (TP), thus overcoming the barriers to the application of miR-497: low transcriptional efficiency, chemical instability, severe systemic toxicity and weak aqueous solubility of TP. It was experimentally demonstrated that these nanoparticles were taken up by cisplatin-resistant ovarian cancer cells and significantly enhanced the apoptosis of tumor cells. The mechanism of action may be that the overexpression of miR-496 promotes the dephosphorylation of components of the overactivated PI3K/AKT/mTOR signaling pathway, which in turn facilitates the transition of TAMs from the M2 phenotype to the M1 phenotype and increases the killing effect of TP on cisplatin-resistant cell lines.^[133]^

## 6. Summary and outlook

TAMs are important components of the TME and play important roles in the regulation of tumor cells and the TME. Chemokines secreted by various components of the TME can act on monocytes in the circulatory system, recruiting them and transforming them into TAMs, which are involved in tumor development. TAMs in the TME have 2 phenotypes with distinct functions: M1 TAMs inhibit tumor growth, whereas M2 TAMs promote tumor growth. Recent studies have shown that various components of the TME, particularly tumor cells, can regulate the phenotypes of TAMs through the secretion of various cytokines and exosomal noncoding RNAs. TAMs within the TME are likewise a double-edged sword; when they are stimulated by cytokines and other substances secreted by tumor cells, they undergo M2 polarization to promote tumor progression. M2 TAMs can also act on tumor cells by secreting substances such as cytokines and exosomal noncoding RNAs to promote tumor growth and distant metastasis as well as by inducing chemoresistance and endocrine resistance in tumor cells; M2 TAMs can also accelerate tumor progression by forming a positive feedback loop with tumor cells. However, if we can promote M1 polarization of TAMs within the TME and change the ratio of M1 TAMs to M2 TAMs within the TME, we can change the TME and transform it into a microenvironment that inhibits tumor growth. In recent years, researchers have discovered that some drugs are likely to exert an inhibitory effect on tumor growth by inducing the M1 polarization of TAMs and altering the ratio of M1TAMs to M2 TAMs within the TME. Therefore, a better understanding of the signaling pathways involved in the polarization of TAMs within the TME is important to guide the future development of tumor therapy. In this review, we focused on the role of the AKT signaling pathway in the polarization, autophagy, apoptosis, and infiltration of TAMs within the TME as well as in the crosstalk between TAMs and tumor cells. In addition, we highlight the positive feedback loops formed between tumor cells and TAMs that have been discovered in recent years; these feedback loops can further promote tumor growth and metastasis as well as drug resistance mediated by M2 TAMs. Therefore, there is an urgent need to further elucidate the roles and mechanisms of signaling pathways in the regulation of TAM polarization and intercellular crosstalk within the TME. The activation, inhibition, and interaction of these signaling pathways as well as the blockade of positive feedback between tumor cells and TAMs could lead to promising new targets for tumor-targeted therapy.

## Author contributions

**Writing – original draft:** Changming Liang, Song Wang, Chengwei Wu, Jiawei Wang, Lishuai Xu, Senlin Wan, Xu Zhang.

**Writing – review & editing:** Yinfen Hou, Yabin Xia, Li Xu, Xiaoxu Huang, Hao Xie.
